# Microfoundations of sensing capabilities: From managerial cognition to team behavior

**DOI:** 10.1177/14761270221142959

**Published:** 2022-12-14

**Authors:** Jean-François Harvey

**Affiliations:** HEC Montréal, Canada

**Keywords:** construal level, dynamic managerial capabilities, environmental scanning, sensing capabilities, team learning

## Abstract

Scanning the environment for information about competitors, technology trends, or customer needs allows firms to sense opportunities and threats, which supports dynamic capabilities and helps firms remain competitive over time. There has been significant theoretical development on the cognitive antecedents of dynamic capabilities—so-called *dynamic managerial capabilities*. In this study, I propose a novel mechanism through which managerial cognition can scale to a collective level in support of sensing capabilities and consider how organizational design may influence this relationship. Specifically, I posit that high-construal managers engage in more environmental scanning than low-construal managers do, because their mental horizons are broader and encompass further alternatives, and that over time their behavior is modeled by their team. I also suggest that managers’ degree of task-related interdependence with peer managers across the firm influences the direction of this relationship, with low interdependence reversing it. I find support for my theory using multiple-source, time-lagged data gathered from 88 managers and their team, thereby offering key implications for theory and practice.

Dynamic capabilities—the firm’s capacity to sense opportunities, seize them, and reconfigure itself to profit from them (Teece, 2007)—are vital, as business is increasingly volatile and fraught with unexpected environmental contingencies (Helfat et al., 2007; Teece et al., 2020). Ever-changing consumer needs and competitor activities threaten the profit streams of incumbent firms, whereas emerging technological trends may be difficult to discern. Thus, how firms can develop dynamic capabilities has been central to strategy scholarship, with learning at the heart of it all and particular emphasis on how knowledge is absorbed, processed, and retained (Di Stefano et al., 2010; Zollo and Winter, 2002). As a part of these efforts, much attention has been paid to managerial cognition (Adner and Helfat, 2003; Gavetti, 2005; Helfat and Martin, 2015). *Sensing* capabilities, for instance, demand that managers gain insights from scanning the environment using superior skills in perception and attention—a subset of so-called *dynamic managerial capabilities* (Helfat and Peteraf, 2015). The theory posits that firms must count on managers who can discover new business models or technological trends and recognize incompatibilities between the firm’s strategy and the market while remaining alert to competitor threats (Teece, 2007).

However, the direct connection that is drawn between managerial cognition and sensing capabilities generally overlooks the pivotal role that teams play in acquiring information from beyond the boundary of the firm (see Harvey et al., 2022, for a review). Steve Jobs surrounded himself with teams who could execute his strategy, but those teams also generated unique knowledge that was crucial to Apple’s success (Gladwell, 2011). There are numerous cases of managers who rely on a network of high-performing teams to innovate (e.g. Edmondson and Harvey, 2016; Pisano and Shulman, 2018), and research has shown that managers need the coordinated work of teams to enable the development of dynamic capabilities (Argote and Ren, 2012; Martin, 2011). Yet the manager-team relationship has not been explicitly depicted in the literature. As a result, we know little about whether and how dynamic managerial capabilities come to influence the team-level behavior at the source of the firm’s sensing capabilities.

In this article, I aim to contribute to the microfoundations of sensing capabilities by examining how managers’ cognitive makeup influences their environmental scanning behavior, and, in turn, affects the environmental scanning behavior of the team they manage. By virtue of their position in the hierarchy, managers are a key source of influence over teams (Kozlowski and Ilgen, 2006), playing a central role in constituting socially shared systems for cognition and information processing (Cannon-Bowers et al., 1993). Because of this, managers’ behavioral patterns can be transferred to, and emulated by, team members (Bandura, 1977, 1986). Consequently, managers’ distinct characteristics and behaviors are likely to affect the behaviors that teams adopt (Harvey et al., 2019). Therefore, if managers’ cognitive makeup influences their behavior (Helfat and Peteraf, 2015), I argue that it is also likely to influence the behavior found in teams, and I develop theory about the relational nature of such role-modeling, making the duration of the relationship (i.e. manager tenure) particularly relevant.

Regarding managers’ cognitive makeup in the context of sensing capabilities, I consider their construal level, because it is likely to affect their view of problems and the range of alternatives considered to address them (Gavetti and Levinthal, 2000; Levinthal, 1997). Low-level construals are concrete and detailed, which helps individuals navigate the here-and-now, whereas high-level construals are broad and abstract, which helps individuals consider situations that are distant temporally, spatially, socially, or ontologically (i.e. hypothetical) (Trope and Liberman, 2010). Building on recent management research that investigates construal level as a relatively stable characteristic (Wiesenfeld et al., 2017), I contrast high-construal and low-construal managers (i.e. those who tend to use high-level and low-level construals, respectively). I expect the former to engage further in environmental scanning, because of the big-picture schema they apply to problems.

I also consider managers’ task-related context, because there are significant contextual pressures on managers to focus on the here-and-now (Levinthal and March, 1993; March, 1991), such that task incentives and structure may extinguish the cognitive impetus to engage in environmental scanning (Pisano, 2019; Teece, 2007). I argue that cross-unit interdependence—a structural feature that can generate or limit opportunities for managers to collaborate with peers from different units (Nadler et al., 1975; Puranam et al., 2012; Thompson, 1967)—expose managers to beliefs that may diverge from their own. Given their power of abstraction and analogical reasoning, high-construal managers in such settings are likely to generate even richer sets of alternatives when facing problems and engage in more environmental scanning (Gavetti et al., 2005; Huber, 1991). Conversely, I argue, high-construal managers under low levels of cross-unit interdependence will allow their big-picture representations to ossify (Vallacher and Wegner, 1987, 1989), making the set of relevant alternatives to consider more automatic and increasingly difficult to change (Hodgkinson and Healey, 2011), thereby hindering environmental scanning behavior.

I tested—and confirmed—my hypotheses (see Figure 1) in a time-lagged, survey-based study of 88 managers and their teams. In doing so, I make several contributions. First, I contribute to the microfoundations of dynamic capabilities by investigating an interaction between the cognitive and organizational design antecedents of dynamic capabilities (Helfat and Martin, 2015; Schilke et al., 2018). I show that cross-unit interdependence can provide conditions in which the benefits of managerial cognition can be realized, which augments the relevance of communication and coordination mechanisms across the firm for sensing capabilities (Teece, 2007). Second, I contribute to further understanding of construals in strategy by revealing how high-level construals can become a source of inertia. Research has been overwhelmingly focused on the positive behavioral outcomes of high-level construals, and largely ignores their potential detrimental effects. By theorizing the potential stickiness of high-construal managers’ mental representations (Vallacher and Wegner, 1987, 1989), my study aligns with previous research on managerial cognition (Gavetti and Levinthal, 2000) and opens a new stream of work on construals in strategic management.

**Figure 1.**
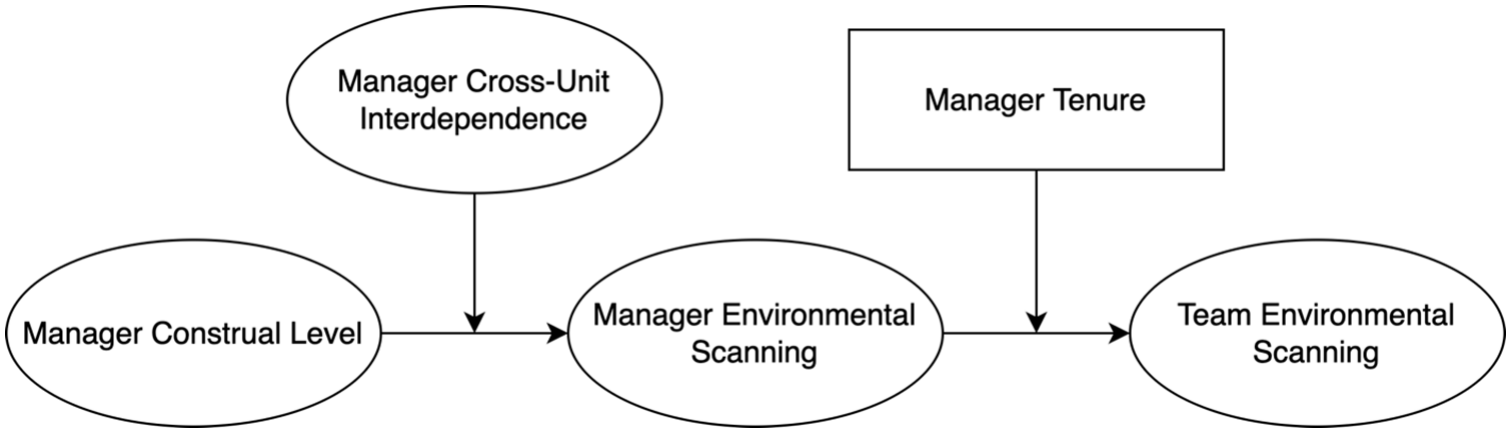
Theoretical model.

Finally, my study sheds light on the mechanism through which managers’ cognition influences behavior at a collective level. Scholars of dynamic capabilities generally agree that the learning that occurs in teams can influence superior firm performance (Harvey et al., 2022). However, research on dynamic managerial capabilities has focused on managers’ cognition and their *own* strategic agency, stopping short of exploring its influence on behavior in teams (e.g. Helfat and Peteraf, 2015). My study helps explain the process by which managerial cognition comes to influence environmental scanning at the team level, and therefore, adds an important theoretical underpinning to the stream of research on dynamic managerial capabilities.

## Theory and hypotheses

I situate my work within the dynamic capabilities framework, which emphasizes the development and renewal of resources regarded as (mostly intangible) firm-specific assets that are difficult to imitate or otherwise replicate (Helfat et al., 2007; Teece et al., 1997). Firms move from intention to outcome by deploying routines (Nelson and Winter, 1982), and the ability to reliably perform and adapt these routines best describes the firm’s dynamic capabilities (Helfat and Winter, 2011; Winter, 2003). Capabilities develop over time through learning (Teece, 2009; Winter, 2000; Zollo and Winter, 2002); knowledge is developed throughout the firm with the purpose of doing useful things, and sometimes transforming what is done, or how (Cohen et al., 1996; Pisano, 1996, 2000). Learning tends to occur in the teams that populate the firm because individual team members’ work-related interactions among themselves can enable them to make sense of complex situations and attain insights that they cannot reach on their own (Edmondson, 2002; Harvey et al., 2019; Senge, 1990).

One key input for learning to occur and for capabilities to develop is the information that is acquired from the environment (Danneels, 2008; Teece, 2007; Teece et al., 1997). Through environmental scanning, firms can gain a superior understanding of the environment by perceiving signals that other firms can miss (Aguilar, 1967). Doing so can, therefore, support strategic decision-making and help secure or improve the firm’s position (Choo, 2001; Duncan, 1972). While our knowledge of environmental scanning largely stems from research on executives (e.g. Daft et al., 1988; Hambrick, 1982; Keegan, 1974), Ancona and Caldwell (1992a) have shed light on the role of teams in enabling new information to enter the firm: team members engage in boundary-spanning behavior that involves “general scanning for ideas and information about the competition, the market, or the technology” (Ancona and Caldwell, 1992a: 641). Teams can pinpoint novel or high-potential technologies, unmet customer needs, and shifts in customer preferences, as well as the threat of innovative product launches by both new market entrants and existing competitors (see also Bresman, 2010). This may involve attending fairs and trade shows, collaborating with university research centers, talking to industry gurus, mingling with early adopters, or getting involved in online communities (e.g. Harvey et al., 2015). The goal is to capture real-time information that can then be processed within the team to produce insights that help the firm update its current routines and perform better, or transform its routines by developing new products, services, or processes (Harvey et al., 2022).

Consider the team of designers at Samsung who generated insights into the world of home entertainment. They produced valuable knowledge for the firm by realizing that, in most homes, TVs were switched off far more than they were switched on. This insight influenced the strategic decision to improve the visual appeal of the firm’s TV products, which had a major impact on the market (Yoo and Kim, 2015). In another example, Swatch needed to recapture market share lost throughout the 1970s to Japanese watch companies (Raffaelli, 2019). It succeeded by uncovering a previously unknown market need. Before Swatch’s innovation, most watches were either affordable and disposable items or expensive jewelry that was treasured for a lifetime. A team of engineers and marketers spoke to consumers and revealed a need for low-cost, high-tech watches that would be bought and worn in multiple styles to match the season or the user’s mood or outfit that day. Treating the watch as a fashion accessory implied owning more than one at a time and frequently adding to the collection—just as fashion-conscious consumers did with shoes. With regard to new technologies, today’s financial firms need their teams to keep abreast of digital technologies like artificial intelligence, robotics, and blockchain in case their industry is suddenly disrupted—just as the telecom industry was disrupted by products offering free digital telephony such as Skype and WhatsApp (Edmondson et al., 2018). Such challenges are now present in most industries (see Dionne and Carlile, 2019 for the example of healthcare).

As a construct, environmental scanning contrasts with internal learning, which concerns team members’ own experiences, such as seeking feedback, sharing information, and discussing errors (Wong, 2004). The literature is also ripe with evidence showing that teams can improve their performance through internal learning (Leblanc et al., 2022). However, although internal learning may allow the team to learn about its work, it is also possible that team members may lack the knowledge or prior experience required for a particular task (Bresman, 2010). Also, the knowledge and expertise within the team may not always represent the most recent or relevant information about the competitive landscape in which the team operates (Hargadon and Sutton, 1997). In contrast, learning from the environment may provide the team with more opportunities to learn about its work over and above those provided by internal learning activities alone (Ancona and Caldwell, 1992b; Harvey et al., 2022).

Teams are, therefore, vital for firms to keep in step with their environment. Of course, teams are profoundly influenced by managers (Edmondson, 1999), whose behavior tends to be modeled by team members (Bandura, 1977, 1986). Given that mental processes—namely, those for attention and perception—are especially important for managers’ own environmental scanning (Helfat and Peteraf, 2015; Hodgkinson and Healey, 2011), I first consider the way managers think about the world to explain which teams engage most frequently in environmental scanning themselves.

### Construal-level theory

Construal-level theory centers on the degrees of abstraction in our mental representations of the world (comprising “targets” such as things, people, events, and activities) and their consequences for judgment and behavior (Trope and Liberman, 2010; Wiesenfeld et al., 2017). The common expression “seeing the forest for the trees” captures the distinction between low-level construals, which are concrete and detailed (i.e. seeing the trees), and high-level construals, which are abstract and schematic (i.e. seeing the forest). For example, one could mentally represent smartphones as either “the iPhone 14” or at a higher level as “a platform business model.” When people think of their goals and their related activities, lower-level construals narrow the attention to specific means (i.e. *how* things are done) while higher-level construals broaden the attention to overarching goals (i.e. *why* things are done). Continuing with the smartphones example, the lower-construal individual would want to understand the intricacies of all the tasks the device can perform, while the higher-construal one would ponder the effects it has on business and society.

The theory posits that our direct, human experience is limited to what is happening to us in the here-and-now; everything else is mentally constructed (Trope and Liberman, 2010). Through these mental constructions, we can think about situations that happened in the past, will happen in the future, might happen, are happening elsewhere, or happen to other people. Extensive research has shown that higher-level construals are associated with greater psychological distances in people’s judgment and behavior (Henderson et al., 2011; Trope and Liberman, 2010). High-construal people pay more attention to the desirability of end-states in their decision-making, while low-construal individuals narrow it down to the feasibility of specific means (Liberman and Trope, 1998). High-level construals also lead people to give more weight to idealistic values in their decisions, whereas low-level construals increase the salience of pragmatic concerns (Kivetz and Tyler, 2007). In an experiment on business performance assessments, high-construal participants focused more on long-term, general trends, while low-construal participants directed their attention to short-term, local deviations (Nussbaum et al., 2006).

Reyt and Wiesenfeld (2015) brought attention to the level at which employees construe their work role to investigate the interplay between employees’ mental representations, their work environment, and their downstream behavior. Given that social roles (e.g. “manager”) have relatively set characteristics, the authors studied role construals as stable domain-specific constructions that can predict patterned behavior. This line of research is a departure from traditional construal-level research, which is mainly laboratory-based and focuses on construal level as a state (e.g. Fujita et al., 2006). Yet, studies involving repeated construal-level measures confirm the stability of people’s construals over several months (Reyt and Wiesenfeld, 2015; Vallacher and Wegner, 1987), and I adopt this trait-like conceptualization in my theorizing.

### Construal level and environmental scanning

As noted earlier, environmental scanning is about searching for information about competitors, technology trends, or customer needs outside of the firm’s boundary. Although such scanning is vital for firms to develop their capabilities to achieve superior performance (Teece, 2007; Teece et al., 1997), managers have reported engaging surprisingly little in the behavior (Haase and Franco, 2011; Keegan, 1974). This can be explained by the fact that managers generally form mental models that enable them to find solutions efficiently by simplifying the issues they encounter to bring them within the bounds of their processing power (March and Simon, 1958; Simon, 1991; Walsh, 1988). Specifically, managers consider their “performance landscape”—that is, the number of distinct attributes that must be addressed in a given problem (e.g. production, branding, distribution, etc., when launching a new product) and how the payoff of getting each attribute right depends on one or more other attributes (Levinthal, 1997). Based on the alternatives they can think of for each attribute (e.g. producing in-house vs outsourcing production), managers choose to engage in certain behaviors, make decisions, and glean information from their consequences. Over time, they can further define their performance landscape, making their behaviors and decisions increasingly “automatic.” Managers’ performance landscape is a simplification of the actual landscape—the many attributes, interrelationships, and alternatives that *could* be considered—which affords them the agency they need to deal regularly with multiple problems (Gavetti and Levinthal, 2000).

Over time, managers, therefore, tend to engage only minimally in environmental scanning, because doing so rarely helps to simplify problems; instead, it tends to make their performance landscape richer and more complex (Csaszar and Levinthal, 2016; Martignoni et al., 2016). Indeed, environmental scanning imposes significant information-processing demands, such as merely what to pay attention to, or how to integrate what is found into one’s performance landscape in a manner that supports decision-making (Cohen and Levinthal, 1990; Daft and Weick, 1984). As a result, managers tend to direct their attention toward what is familiar, convenient, or less demanding (Cyert and March, 1963), and will only look beyond the firm’s boundary and engage in environmental scanning if they perceive that it is absolutely necessary (Helfat and Peteraf, 2015; Salancik and Pfeffer, 1978). This begs the question of which managers perceive such a necessity and are motivated to extend themselves in such a manner—and construal level may be a significant part of the answer.

Construal level offers a useful perspective on environmental scanning behavior because cognitive abstraction is a vital factor in the analogical reasoning process that leads individuals to seek information outside their immediate task environment (Huber, 1991). Managers’ construal levels can, therefore, make environmental scanning more or less natural for them. The performance landscape that managers develop will reflect their construal level: higher-construal managers form mental models that cover more mental ground than those of lower-construal managers (cf. Vallacher and Wegner, 1989). The former instinctively consider broader mental horizons across all dimensions of psychological distance (i.e. temporal, spatial, social, hypotheticality) (Reyt and Wiesenfeld, 2015). Thus, they will likely develop wider, or more extensive, performance landscapes as they envision more long-term or distal consequences than lower-construal managers (Mueller et al., 2014; Rim et al., 2013), making them more likely to expand their search for information beyond the here-and-now (Gavetti et al., 2012; Newell and Simon, 1972). Lower-construal managers, for their part, are much more immersed in idiosyncratic details and concerns over feasibility (Mount et al., 2021). They tend to expand their view only when there is a specific, novel issue to address (Park and Baer, 2022); their narrower mental models generate fewer need areas—a less extensive performance landscape—that require new information only when things are not working satisfactorily (cf. Levinthal and March, 1993).

Furthermore, managers’ construal levels can make it more or less demanding to engage in environmental scanning. Higher-level construals can lead managers to pay attention to abstract and decontextualized information to fit their performance landscape, whereas managers construing the world at lower levels pay attention to more concrete information (Wakslak and Trope, 2009). Yet concrete information—in contrast to abstract information—includes many more incidental features and peripheral characteristics (Trope and Liberman, 2010). Given that managers aim to conserve their information-processing power (Simon, 1991), lower-construal managers are likely to need more motivation to invest time and energy in environmental scanning than their higher-construal counterparts. Taken together, I hypothesize the following:

*Hypothesis 1.* Managers’ construal level is positively related to their environmental scanning behavior.

### Moderating role of cross-unit interdependence

Construal level is not the only factor that can influence the cognitive processes of perception and attention at the heart of environmental scanning; social interactions also help spread beliefs and behaviors among individuals (Bandura, 1977; Gavetti and Levinthal, 2000). Features of organizational design that stimulate social interactions may thus shape managers’ performance landscape in ways that affect their search for information beyond the firm’s boundary. They may also influence the effect that managers’ construal level has on their scanning behavior.

I focus on managers’ task-related interdependence with managers from other units of the firm, which represents the degree to which a unit’s performance depends upon the efforts or skills of other units (Nadler et al., 1975; Puranam et al., 2012). Such interdependence stems from a workflow in which units rely on each other to achieve goals: it can be very low, such as when one unit has all the resources necessary to complete its job, or it can be very high, such as when the successful completion of a job depends on the input of several units (see Thompson, 1967 on reciprocal interdependence). Task-related interdependencies across units can be manipulated through organizational design to generate information-processing requirements (Galbraith, 1973; Mohr, 1971). Managers under higher degrees of interdependence expend greater effort on communication and joint decision-making (Adler, 1995; Gavetti et al., 2007; Hambrick et al., 2015; March and Simon, 1958). Classic examples are when marketing, engineering, and production units work toward the development of a new product, or when risk and compliance, sales, and information technology units are involved in implementing new software for managing customer relationships.

I argue that managers’ degree of task-related, cross-unit interdependence can influence whether their higher-level construals translate into more environmental scanning behavior. Managers who collaborate with peers from different units to make decisions are likely to discover other performance landscapes: different attributes to consider in a problem, unexpected interrelationships, and unknown alternatives that must be considered (Gavetti et al., 2005). Indeed, if one manager’s performance landscape constitutes a simplified caricature of their own decision context (Gavetti and Levinthal, 2000), then adding managers from other units to the decision context can turn the caricature into a more rounded portrait (Martignoni et al., 2016). High-construal managers are more likely to engage in cross-unit collaboration in mindful ways, rather than simply addressing the given problem at hand and moving on (Levinthal and Rerup, 2006). Their abstraction capacity—the essence of analogical reasoning (Gick and Holyoak, 1983; Reeves and Weisberg, 1994)—can lead them to transfer learnings to other situations and thus enhance their performance landscape when facing problems (Gavetti et al., 2005). Consider operations managers who must collaborate with marketing managers and learn how online communities are used to gather key insights. High-construal operations managers may decontextualize such learnings and apply them to the very problems they are facing. Put differently, richer performance landscapes are likely to push high-construal managers to shape perception and attention processes toward further environmental scanning (Gavetti and Levinthal, 2000; Helfat and Peteraf, 2015).

High construals, however, may backfire when managers have little reason to collaborate with peers from other units. As described by Gavetti and Levinthal (2000), while the performance landscape may be extensive, the factors that are ultimately considered can become a consistent set of behaviors over time—represented by the “peaks” of the landscape. The authors argue that if managers have few social interactions with their peers, they can form the belief that they have figured out the ideal factors to consider when they encounter problems in their own, very stable decision context. This is akin to the myopic tendencies that limit managers’ ability to adapt their mental models even when courses of action are changing significantly (March, 1991; Miller, 1990; Tripsas and Gavetti, 2000). Once their decision factors have crystallized in this way, managers may gravitate toward what is most familiar and focus their learning inwards, overlooking more distant times, places, and experiences (Levinthal and March, 1993). High-construal managers with little required interdependence, I argue, are subject to such “learning myopia.” Indeed, it has been argued that high-construal individuals can develop mental models that become particularly “sticky” or hard to change (Vallacher and Wegner, 1987, 1989). It can even feel “painful” for them to venture outside their already-formed mental models (Hodgkinson and Healey, 2011). Therefore, taken together, I propose:

*Hypothesis 2.* The relationship between managers’ construal level and environmental scanning behavior is moderated by manager interdependence, such that the relationship is positive (vs negative) when manager interdependence is high (vs low).

### From manager to team behavior

Managers have a strong influence on how employees conduct themselves (i.e. social-learning theory; Bandura, 1977, 1986; Manz and Sims, 1981). Because of their lower status in the hierarchy, subordinates typically scrutinize managers to learn which behaviors are appropriate, and which are undesirable. For instance, managers act as role models by reflecting on work processes and discussing errors or near-misses, which stimulates their subordinates to engage in the same internal learning behaviors (Edmondson et al., 2001; Hu et al., 2018; Nembhard and Edmondson, 2006). Research even corroborates that by interpreting managers’ emotional cues, team members emulate their affective and motivational states at work (Dragoni and Kuenzi, 2012; Johnson, 2008; Owens and Hekman, 2016).

However, given that environmental scanning behavior largely takes place outside the immediate team environment, team members may not be able to watch their manager finding out what competing firms are doing, or searching for new, useful technologies. Hence, it may be practically difficult for team members to perceive whether and to what extent their managers engage in environmental scanning. Little empirical research has considered whether social-learning theory applies to target behaviors that occur outside the emulators’ immediate task environment. Recent theoretical developments on a relational view of role-modeling can offer guidance here: social learning best occurs through a two-way interaction where role model and learner intentionally share and co-construct meaning about a given behavior (Harvey, 2012; Myers, 2018). Similarly, construals have also been shown to impact nearby individuals through interaction and verbal exchange (e.g. Reyt et al., 2016).

Specifically, managers who engage in environmental scanning do not merely acquire facts about technological or business trends; they also acquire “the ability to act in the world in socially recognized ways” (Brown and Duguid, 1991). Through their environmental scanning, they develop a specific role identity that is revealed through the discourse that they craft for themselves and for those around them (Ashforth et al., 2008). The degree to which managers focus on the external environment may crystallize into a particular identity that managers also project on to their team with the issues that they put forward for their team to examine. By talking with their team about a new technology discovered at a trade show, or a new business model described in a market report, managers provide influential input about what is of value in the team (Zaccaro et al., 2001). The team is then more likely to develop a stronger external focus, which has been shown to promote environmental scanning behavior among team members (Marrone et al., 2007). Therefore, I posit:

*Hypothesis 3*. Manager environmental scanning behavior positively influences team environmental scanning behavior.

Individuals mimic those close to them more than strangers (Bandura, 1986), indicating that the richer interpersonal interactions found in enduring relationships can facilitate role-modeling. Therefore, I consider the tenure of the manager with the team because pre-existing rapport likely influences the trickle-down effect of the manager’s behavior; team members’ mimicry of their manager can be influenced by their shared history of interaction (e.g. Gardner et al., 2012; Gruenfeld et al., 1996). The more exposure team members have to the manager, the more powerful social learning from them will be (Myers, 2018). Thus, I propose:

*Hypothesis 4*. Manager tenure positively moderates the relationship between manager environmental scanning behavior and team environmental scanning behavior, such that manager environmental scanning behavior has a strong positive effect on team environmental scanning behavior when manager tenure is high, but not when it is low.

## Methods

### Participants and procedures

I collected data from a North American firm in the mortgage industry, consisting of approximately 2000 employees and with annual revenues of USD 5 billion. I surveyed all managers within the firm and all their subordinates. Most employees were professionals holding at least a bachelor’s degree, and they were distributed across 268 teams. I invited all team members and managers to complete surveys at two different points in time. First, I asked managers about their work environment and the mental representations of their job (T1) and, 8 weeks later, I explored both managers’ and team members’ behavior (T2). All surveys were strictly voluntary and confidential, and I used non-identifying codes to aggregate data across the surveys and to conduct my analysis. A total of 251 (T1; 94% response rate) and 198 (T2; 74% response rate) managers and 768 (T2; 51%) team members completed my surveys. I used the sampling theory suggested by Dawson (2003) to identify a cutoff participation rate for each team based on its size (e.g. three out of seven), yielding a total of 88 teams for analysis (33% of the initial sample). I tested for possible response bias in my data by running a series of analyses of variance (ANOVAs). I compared my final sample with (a) the 251 managers who responded to T1 only, and (b) the 198 managers who responded to T2 only, but for whom I did not received team-level data that fit my minimum participation rate for aggregation. In both cases, the results suggested no significant mean differences on my focal variables.

### Measures (see Appendix 1)

#### Manager construal (T1)

I measured managers’ construal level using nine items from Reyt and Wiesenfeld’s (2015) Work-Based Construal Level (WBCL) scale. The WBCL includes 18 common work activities (e.g. “developing a budget”), each characterized by a low-level description focused on *how* things are done (e.g. “listing expenses and revenues”), and a high-level description focused on *why* things are done (e.g. “managing funds”). For each activity, high- and low-level descriptions serve as opposite anchors of 7-point scales, and participants indicate the point that best describes the activity for them. The WBCL was developed as a work-specific adaptation of Vallacher and Wegner’s (1989) Behavior Identification Form, which included 25 non-work activities, and is one of the most widely used measure of construal level (Burgoon et al., 2013). The temporal reliability of both measures was demonstrated over several months (Reyt and Wiesenfeld, 2015; Vallacher and Wegner, 1989). I selected nine items with my contacts at the participating firm, based on their relevance to the managerial position at their firm. The activities I used were preparing a report, obtaining information from someone, assigning work to someone, communicating information to someone, attending a meeting, developing a procedure, hiring someone, developing a budget, and evaluating someone’s performance. Two items were reverse-scored, and responses were averaged to construct the measure (α = .87).

#### Manager interdependence (T1)

I adapted the four-item scale from the Michigan Organizational Assessment Questionnaire (Nadler et al., 1975) to measure manager interdependence. Items are “Managers from different units do not need each other to make decisions,” “It is necessary for managers from different units to work together to get the job done,” “It is in my best interest for other managers to perform well,” and “What other managers do affects what I can do.” Items were measured with a 7-point Likert-type scale (1 = strongly disagree; 7 = strongly agree), one item was reverse-scored, and responses were averaged to construct the measure (α = .80).

#### Manager environmental scanning (T2)

I adapted the four-item scale from Ancona and Caldwell (1992a) to measure manager environmental scanning behavior. I shifted the referent from “We” to “I” to better capture the individual-level variable (Kozlowski and Klein, 2000). Items included: “I look for what competing firms are doing,” “I scan the environment for market ideas/expertise,” “I collect technical information/ideas from individuals outside my firm,” and “I scan the environment for technical ideas/expertise.” Again, items were measured with a 7-point Likert-type scale (1 = strongly disagree; 7 = strongly agree), and responses were averaged to construct the measure (α = .86).

#### Manager tenure (T2)

I asked managers responding to the second survey for how long they had been managing their current team (in years) to measure manager tenure. The data range from 0.5 to 10 years. The average is 3.31 years (SD: 2.65) and data distribution is slightly skewed (1.23) and kurtotic (0.69).

#### Team environmental scanning (T2)

I used the four-item scale from Ancona and Caldwell (1992a) to measure team environmental scanning behavior. I prompted participants to reflect on their own behavior and the behavior of their fellow team members, and I used the “We” referent to capture the team-level variable (Kozlowski and Klein, 2000). Items included: “We look for what competing firms are doing,” “We scan the environment for market ideas/expertise,” “We collect technical information/ideas from individuals outside our firm,” and “We scan the environment for technical ideas/expertise.” Items were measured with a 7-point Likert-type scale (1 = strongly disagree; 7 = strongly agree), and responses were averaged to construct the measure (α_team_ = .89).

#### Control variables

I controlled for several variables that have been shown to have an important effect on an individual or team behavior. I controlled for managers’ age (actual age) and gender (male = 0; female = 1). Managers may process leadership roles differently depending on their age or gender (e.g. Eagly, 1987), and team members may use age or gender to draw interpersonal inferences about their managers, influencing how managers’ behavior influence their subordinates (Hentschel et al., 2019). I also controlled for team size (number of members) because larger teams may have more task-relevant resources that reduce the need for environmental scanning (Stewart, 2006). Moreover, I controlled for team tenure (number of years) because teams that have no personnel reshuffles for an extended period tend to display lower levels of environmental scanning (Katz, 1982). Finally, I controlled for the organizational location of the teams studied (Branch = 0; Headquarter = 1) and the nature of the tasks in which they engage (Operations = 0; Functional = 1). The organizational location may influence environmental scanning behavior because of the amount of accessible resources (Leiponen and Helfat, 2011), and the tasks may make environmental scanning more or less natural (Harvey et al., 2022).

### Validity and aggregation of survey data

#### Internal validity

My survey included 21 items comprising four constructs across T1 and T2, and I performed a confirmatory factor analysis (CFA) to verify the validity and distinctiveness of the measures. I modeled all items under their respective latent factor, and goodness-of-fit indices show satisfactory fit for a four-factor model of the data: χ²(183) = 204.127 (p = .136), comparative fit index (CFI) = .973, Tucker–Lewis index (TLI) = .969, root mean square error of approximation (RMSEA) = .036, and standardized root mean square residual (SRMR) = .071. I tested an alternative three-factor model where *manager environmental scanning* and *team environmental scanning* were modeled under the same latent variable. The chi-square difference test provides evidence for my hypothesized four-factor solution (Δχ² = 177.05, Δdf = 3, p < .001).

I also tested for reliability as well as convergent and discriminant validity using each factor’s average variance extracted (AVE) and composite reliability (CR). The AVE values are .44 (manager construal level), .51 (manager interdependence), .63 (manager environmental scanning), and .66 (team environmental scanning), and the CR values range from .80 (manager interdependence) to .89 (team environmental scanning). While one AVE value is lower than .50, the CR values are all higher than .70. Most importantly, all AVE values are higher than their respective maximum-shared variance (MSV), and the square root value of each AVE is greater than the correlation value with any of the other latent variables. MSV values range from .07 (manager construal level) to .14 (manager interdependence). The correlation values range from .14 to .26 for manager construal level. Furthermore, I tested for the heterotrait–monotrait ratio of correlations and found it to range from .138 to .409. Therefore, satisfactory reliability as well as convergent and discriminant validity were achieved (Hair et al., 2014).

#### Common method bias

Even though data were collected at different times and from multiple sources (managers and team members), they could still suffer from common method bias because they were all collected using the same tool (surveys). I assessed such possible bias using CFA and modeled a single common latent factor for all items, in addition to the other latent factors. I found that none of the items load significantly on the common latent factor, the factor loadings remain significant on their respective latent factor, and the fit indices are not improved. Specifically, the differences between the loadings with and without the common latent factor range from .036 and .087, and the common latent factor is shown to explain 8% of common shared variance. Based on these results, I felt confident to proceed with my analysis of the data (see Podsakoff et al., 2003).

#### Data aggregation

To test whether it was appropriate to aggregate data on *team environmental scanning* from individual team members to teams, I computed interrater agreement scores (rwg(j)) to test whether the variance of responses within groups was lower than the variance between groups (James et al., 1993; LeBreton et al., 2003). Assuming a normal distribution in responses and using ANOVA to calculate differences in means, I found that the rwg(j) score (.82) was higher than the recommended .70 threshold (George and James, 1993). I then calculated intraclass correlation coefficients (ICC(1) and ICC(2)) to assess the variance explained by team membership and the reliability of team means. I found the following results: ICC(1) = .18, ICC(2) = .46; F = 1.85, p < .001). While the measure for ICC(1) is satisfactory (LeBreton and Senter, 2008), the measure for ICC(2) does not achieve the .60 criterion recommended by some scholars (e.g. Glick, 1985)—although others call this threshold an “arbitrary line in the sand” that must be understood in the context of other factors^1^ (LeBreton and Senter, 2008, p. 835). Notably, ICC(2) reflects the internal consistency of team responses, which are systematically higher in larger groups. Because the teams in my study are relatively small, a low ICC(2) coefficient may not indicate a lack of internal consistency (LeBreton et al., 2003; Mathieu et al., 2020; Shieh, 2016). Therefore, I felt confident in aggregating my data from individuals to teams, particularly because the rwg(j) coefficient is high (Bliese, 2000).

### Analytical strategy

Using the lavaan R package (Rosseel, 2012), I conducted structural equation modeling (SEM; Bollen, 1989) using maximum-likelihood estimation to test my hypotheses. SEM is a useful framework for multivariate hypothesis testing because it allows for multiple equations to be estimated simultaneously (Ullman and Bentler, 2003). Such models comprise latent variables (i.e. variables that were not directly measured, but may be inferred from rated items). In my case, I used each construct’s items to create latent variables—these latent variables represent the common variance between all their respective items. The interaction terms were created using double mean centering as described in Lin et al. (2010). *Manager construal level*, *manager interdependence*, and the latent factor representing their interaction were allowed to freely covary while also predicting *manager environmental scanning*. For its part, *team environmental scanning* was regressed on *manager environmental scanning*, *manager tenure*, and their interaction term, and the latter variables were allowed to freely covary. It was also regressed on *manager construal level*. The fit indices for my hypothesized model with my control variables show good fit to the data: χ²(460) = 505.956 (p = .068), CFI = .952, TLI = .945, RMSEA = .034, SRMR = .078. Thus I used it to consider each hypothesis.

## Results

Descriptive statistics and correlations are shown in Table 1. It is noteworthy that *manager environmental scanning* is positively correlated with *manager construal* (.20), *manager interdependence* (.34), and *team environmental scanning* (.27). The latter is negatively correlated with *team size* (−.22) and *team tenure* (−.20). This indicates that the longer team members have been on the team, and the larger the team is, the less likely the team is to engage in environmental scanning. This is in line with previous research with regard to the effects of both team size (Stewart, 2006) and team tenure (Katz, 1982) on exploratory behavior in teams, allowing greater confidence in my findings.

**Table 1.** Descriptive statistics and bivariate correlations.

	*M*	*SD*	*Min*	*Max*	1	2	3	4	5	6	7	8	9	10	11
*1. Team Size*	7.30	3.28	4	19	–										
*2. Team Tenure*	3.09	2.23	0.27	9.44	0.07	–									
*3. Team Location*	0.56	0.50	0	1	−0.11	−0.43**	–								
*4. Team Task*	0.58	0.50	0	1	0.08	0.19^†^	−0.39**	–							
*5. Manager Age*	41.78	5.93	28	58	0.07	0.03	−0.15	0.20*	−						
*6. Manager Gender*	0.53	0.50	0	1	0.09	0.01	−0.10	−0.10	0.04	−					
*7. Manager Tenure*	3.31	2.65	0.50	10	−0.01	0.02	−0.03	0.13	0.09	0.08	−				
*8. Manager Interdependence*	6.14	0.77	3	7	−0.02	0.06	0.01	0.07	−0.08	−0.05	0.09	(0.80)			
*9. Manager Construal*	5.91	0.80	3	7	0.03	−0.07	−0.03	−0.14	0.07	−0.17	0.02	0.15	(0.87)		
*10. Manager Environmental Scanning*	5.71	0.93	3	7	0.06	0.01	0.18	−0.14	0.13	−0.15	0.12	0.34**	0.20^†^	(0.86)	
*11. Team Environmental Scanning*	5.02	0.83	2.75	7	−0.22*	−0.20^†^	0.26*	−0.03	0.06	−0.18	0.25*	0.11	0.14	0.27*	(0.89)

SD: standard deviation.

N = 88 teams.

† p < .10; *p < .05; **p < .01.

**Figure 2.**
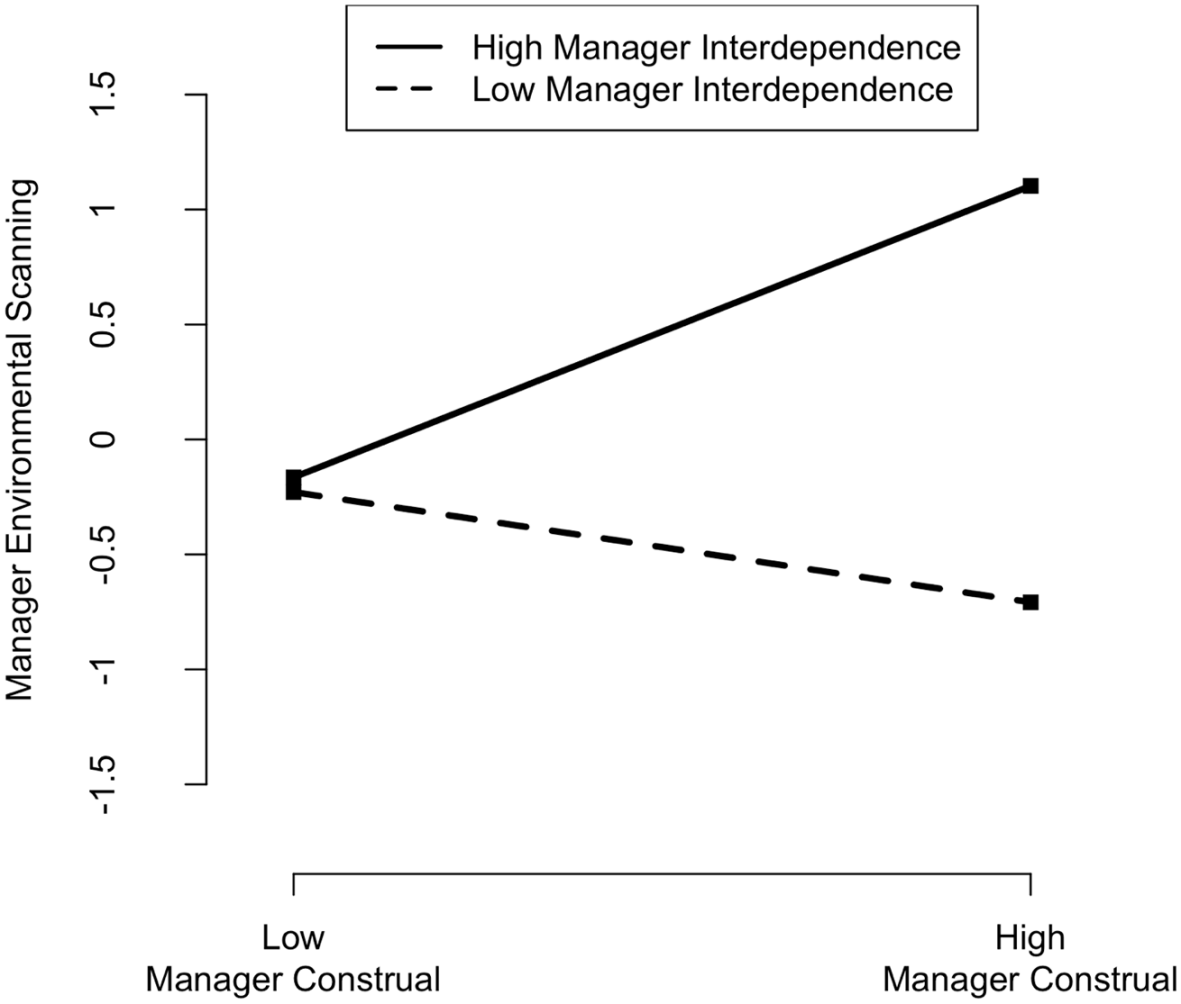
Effect of manager construal level on manager environmental scanning at high and low levels of manager interdependence.

My first hypothesis predicted that the construal level of managers positively influences their engagement in environmental scanning. I found that *manager construal* is positively associated with *manager environmental scanning* (β = .197, p = .038). Therefore, Hypothesis 1 is supported. In my second hypothesis, I posited that higher construals have a stronger influence on the environmental scanning behavior of managers who operate under higher degrees of interdependence. I found that *manager interdependence* is directly associated with *manager environmental scanning* (β = .469, p ⩽ .001), as is its interaction with *manager construal* (β = .437, p ⩽ .001). I plotted and computed slopes at high (1 standard deviation (SD) above the mean) and low (1 SD below the mean) values of the moderating variable. As depicted in Figure 2, results show a negative effect for lower levels (−1 SD) of *manager interdependence* (β = −.240, p = .023), and a positive effect for higher (1 SD) levels (β = .634, p ⩽ .001). Higher-construal managers thrive under higher interdependence, engaging in more environmental scanning, while those same managers neglect environmental scanning when under lower degrees of interdependence. Hypothesis 2 is thus supported.

**Figure 3.**
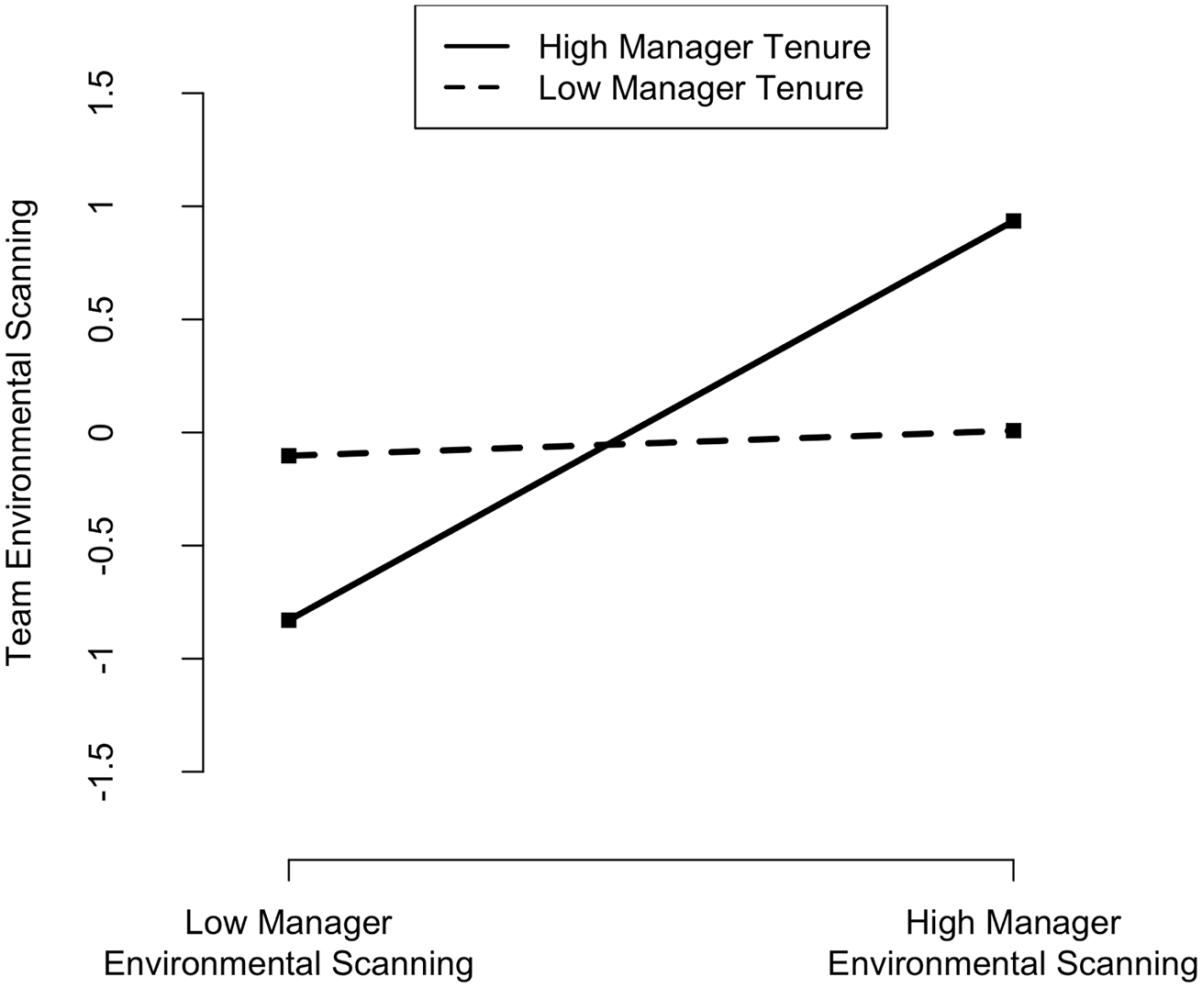
Effect of manager environmental scanning on team environmental scanning at high and low levels of manager tenure.

My third hypothesis suggested that the environmental scanning behavior from managers influences the degree of environmental scanning behavior from their team. I found that *team environmental scanning* is positively associated with *manager environmental scanning* (β = .390, p ⩽ .001). Therefore, Hypothesis 3 is supported. I posited in my fourth hypothesis that the latter relationship is contingent on the team’s exposure to the manager. I found that *manager tenure* is not directly associated with *team environmental scanning*, but that its interaction with *manager environmental scanning* is (β = .411, p ⩽ .001). I plotted and computed slopes at high (1 SD above the mean) and low (1 SD below the mean) values of the moderating variable. As represented in Figure 3, results did not show a significant effect for lower levels of *manager tenure* (−1 SD), but there was a strong significant effect for higher levels (1 SD) (β = .801, p ⩽ .001). This suggests that managers’ environmental scanning behavior takes time to trickle down to the team. Only longer-tenured managers have significant influence, meaning that their engagement in environmental scanning is most contagious as team members model the behavior. Hypothesis 4 is thus supported.

I examined the results of my full model, which suggests that *manager construal* influences *team environmental scanning* via *manager environmental scanning*, and that this indirect relationship is moderated by both *manager interdependence* (first-leg moderation) and *manager tenure* (second-leg moderation). To account for both moderations, I estimated the value of *team environmental scanning* at high, average, and low values of both *manager interdependence* and *manager tenure*, while values of *manager construal* and *manager environmental scanning* were kept constant. Specifically, I used the “:=” operator in the lavaan model syntax and bootstrapping techniques (see Rosseel, 2012). Presented in Table 2, results suggest that longer-tenured managers’ construals can lead to more environmental scanning in teams through their own engagement in environmental scanning when they experience higher degrees of interdependence. On the contrary, the estimate is negative when longer-tenured managers experience lower degrees of interdependence. No significant estimate is found for shorter-tenured managers. This indicates the presence of a chain of effects of *manager construal* →*manager environmental scanning* → *team environmental scanning* that is moderated by *manager interdependence* and *manager tenure*. Although additional research would be needed to investigate this conjecture, it aligns nicely with an overall view of dynamic managerial capabilities such as manager’s cognition influencing the learning that occurs in teams—and explaining the conditions under which this is more likely to happen.

**Table 2.** Estimated value of team environmental scanning under conditions of high, average, and low values of manager interdependence and manager tenure.

Manager interdependence value	Manager tenure value	Team environmental scanning estimate (z value)	95% CI
High	High	0.936 (3.649)	*** [0.419, 1.391]
High	Average	0.430 (2.961)	** [0.151, 0.744]
High	Low	−0.076 (−0.058)	*ns*
Average	High	0.167 (1.923)	^†^ [−0.003, 0.268]
Average	Average	0.077 (1.797)	^†^ [−0.006, 0.137]
Average	Low	−0.014 (−0.058)	*ns*
Low	High	−0.601 (−3.033)	** [−1.053, −0.226]
Low	Average	−0.276 (−2.604)	* [−0.555, −078]
Low	Low	0.049 (0.058)	*ns*

CI: confidence interval.

Values of manager construal and manager environmental scanning are kept constant.

† p ⩽ .10; *p ⩽ .05; **p ⩽ .01; ***p ⩽ .001.

Finally, I ran my models excluding the control variables as a robustness check. I found no improvement in my model, and the changes to the coefficients and their statistical significance are marginal. In other words, all my findings hold with and without controls. The results of my model are summarized in Table 3.

**Table 3.** Results of full model using structural equation modeling.

	Manager environmental scanning	Team environmental scanning
	β (z)	β (z)
*Team Location (Branch* *=* *0; HQ* *=* *1)*	0.276 (2.614)**	0.126 (1.135)
*Team Task (Operations* *=* *0; Functions* *=* *1)*	−0.100 (−1.044)	0.239 (2.337)*
*Team Size*	0.011 (0.129)	−0.160 (−1.759)^†^
*Team Tenure*	0.159 (1.649)^†^	−0.169 (−1.666)^†^
*Manager Age*	0.235 (2.534)*	−0.033 (−0.0346)
*Manager Gender (Male* *=* *0; Female* *=* *1)*	0.108 (1.203)	−0.063 (−0.675)
*Manager Construal*	0.197 (2.078)*	0.146 (1.474)
*Manager Interdependence*	0.469 (4.122)***	
*Manager Construal x Manager Interdependence*	0.437 (3.676)***	
*Manager Environmental Scanning*		0.390 (3.306)**
*Manager Tenure*		0.048 (0.510)
*Manager Environmental Scanning x Manager Tenure*		0.411 (3.913)***
*R²*	0.504	0.483
Goodness-of-fit indices: χ²(460) = 505.956 (p = .068), CFI = .952, TLI = .945, RMSEA = .034, SRMR = .078		

HQ: headquarter; CFI: comparative fit index; TLI: Tucker–Lewis index; RMSEA: root mean square error of approximation; SRMR: standardized root mean square residual.

n = 88 teams; All regression coefficients are based on standardized variables with mean = 0 and SD = 1.

† p ⩽ .10; *p ⩽ .05; **p ⩽ .01; ***p ⩽ .001.

## Discussion

The evolutionary theorists at the roots of the dynamic capabilities framework were skeptical about the degree to which human cognition affects behavior in firms, due to the pervasive influence of organizational rules and routines (Nelson and Winter, 1982). Since then, dynamic capabilities scholars have argued that managers do matter (Augier and Teece, 2009). They have acknowledged the psychological foundations of dynamic capabilities (Hodgkinson and Healey, 2011) and developed theory on dynamic managerial capabilities (Helfat and Martin, 2015; Helfat and Peteraf, 2015). My study sought to contribute to this work by investigating a key facet of managerial cognition—construal level—and a key behavior at the source of sensing capabilities—environmental scanning. Specifically, my study assessed whether managers’ construals can spur their engagement in environmental scanning, and in turn stimulate environmental scanning in teams. It also examined two key boundary conditions of that mechanism—namely, the degree of task-related, cross-unit interdependence experienced by managers, as well as their tenure as leaders of their team. Overall, the results show that high-construal managers positively influence their team’s environmental scanning behavior through their own behavior, and that this indirect relationship is stronger when managers operate under conditions of higher interdependence and when their tenure is longer. In contrast, higher-level construals can backfire under conditions of low interdependence, and managers’ behavior does not scale to the team level when they have only been in position for a short stint. The results of my study offer significant theoretical and practical contributions, in addition to opening important avenues for future research.

### Theoretical and practical implications

My study advances theory on dynamic managerial capabilities and construal level. First, it contributes to the recent stream of strategy research investigating the relevance of construal level (e.g. Mount et al., 2021). In line with its foundation in Action Identification Theory (Vallacher and Wegner, 1989), my conceptualization of construal level as a stable dimension of managerial cognition (cf. Reyt and Wiesenfeld, 2015; Reyt et al., 2016) offers substantial promise in answering questions of interest to dynamic capabilities scholars. My study shows that the difference in cognition can be linked to the strategic agency of managers at the source of sensing capabilities. High-construal managers tend to depart from the familiar, short-term tasks at hand and engage more in environmental scanning than low-construal managers. Although the idea that sensing capabilities are rooted in cognition is not new (Teece, 2007), prior studies have relied on using proxy variables, or simulating the effect of mental models rather than measuring a cognitive attribute directly (Gavetti and Levinthal, 2000; Gavetti et al., 2012). Given that strategic management is “best supported by multiple methods and measures” (Powell et al., 2011: 1371), my study represents a useful step toward further opening the black box of cognition in relation to dynamic capabilities.

Another key contribution to the dynamic managerial capabilities literature relates to the scaling of managers’ cognition to team behavior. Work teams can play a key role in supporting sensing capabilities because the team setting can give rise to reliable patterned behaviors that develop into “environmental scanning routines” (Harvey et al., 2022; see also Cohen et al., 1996). Teams offer a fundamental venue for the self-organized learning from which can emerge new capabilities that help the firm adapt to and/or shape the external environment (Dosi et al., 2000). Obviously, some teams may engage in more strategic learning routines than others (Edmondson, 2002), and my study shows that the manager-team link can explain why that is. My study leverages the ongoing effort to deconstruct dynamic capabilities at the micro level to develop theory on this question, and thus enhances our understanding of the antecedents of dynamic capabilities (Harvey et al., 2022; Schilke et al., 2018). Further integrating social psychology (Bandura, 1977, 1986) with this stream of research, I develop a novel path toward understanding how managers’ cognition can lead to superior firm performance. My study stresses managers’ role-modeling, which provides the conduit through which managers’ cognition can influence firm behavior. My theorizing complements the current focus of dynamic managerial capabilities on managers’ perception and decision-making (e.g. Adner and Helfat, 2003) and helps further connect the stream of research back to the multilevel learning roots of the dynamic capabilities framework (Teece and Pisano, 1994; Teece et al., 1997).

Besides, my study demonstrates that managers can effectively role-model behavior that their teams may not readily observe. Indeed, because environmental scanning occurs outside the team boundary, the more traditional perspective of social learning, which tends to view role models as passive participants in the observation and emulating process (Bandura, 1977; Manz and Sims, 1981), would have encountered limitations in explicating how teams can emulate their managers’ behavior in such cases. Drawing from the relational view of social learning (Harvey, 2012; Myers, 2018), which stresses active, reciprocal interaction between the role model and the learners, I was able to shed light on how managers can shape environmental scanning behavior in their team. This lays the foundation for social-learning theory to be extended to managers’ less discernible behaviors—a vital topic, considering the trend for virtual and remote working prompted by the coronavirus disease-19 pandemic.

Finally, following calls from dynamic capabilities scholars (Helfat and Martin, 2015; Schilke et al., 2018), I consider managers’ cognition with a key feature of organizational design: cross-unit interdependence. In so doing, I find that not all managers with higher construal levels show the same strategic agency. Considering the context in which managers are embedded, beyond their cognition, provides a fuller picture of influence trajectories in their relationship with downstream behavior. My theorizing goes beyond the assumption that abstraction involves greater cognitive flexibility (e.g. Reyt and Wiesenfeld, 2015) to show that context can convert higher construal levels into a source of learning inertia. Since most construal-level research is laboratory-based, context is typically ignored in favor of the identification of main effects. My study demonstrates the importance of leveraging several methods to fully understand the role of construals in strategic management, and it adds to the impetus to consider multiple antecedents of dynamic capabilities to refine theory on dynamic managerial capabilities.

By combining two underpinnings of dynamic capabilities, we also gain a deeper and more sophisticated understanding of organizational design. Specifically, my study extends the literature by illustrating that cross-unit interdependence is more than a mere organizing feature aimed at producing an effective division of labor. By affecting managers’ cognitive representations through continuous knowledge integration, or lack thereof, interdependence becomes a stimulus that influences the evolution of managerial cognition. Future research should further consider how managers’ cognitive makeup matches key features of organizational design. A fruitful related stream of research might be that on strategic human capital, which has considered the question of how individual knowledge, skills, abilities, and other characteristics are amplified by social interactions throughout the firm (Ployhart and Moliterno, 2011). For instance, while the results of my study show that greater interdependence serves to stimulate high-construal managers’ engagement in environmental scanning, future research could consider the units that managers are interdependent with. Managers from units that introduce new technologies—as opposed to more familiar ones—may be those who provide the context for high-construal managers to thrive (Stadler et al., 2022). Expertise markers may also play a key role in the enrichment of managers’ mental representations in cross-unit relationships (Greenwood et al., 2019). These are just two examples of other factors that can be considered in future research to better understand the interplay of cognitive and design factors at the microfoundations of sensing capabilities (see Nyberg et al., 2014).

My study also has several implications for strategy practice. The confirmation that role-modeling is an effective means of shaping collective behavior should be reassuring for managers. They do indeed matter (Augier and Teece, 2009; Helfat and Martin, 2015). To maximize the effectiveness of managers’ role-modeling, firms can draw on my study to provide training that enables managers to be more active in communicating about their experiences with their subordinates. Firms can also leverage the fact that managers’ cognition (i.e. construal level) and the design of their units (i.e. cross-unit interdependence) can influence engagement in environmental scanning. Given that firms may want some groups to engage in this behavior more than others (Edmondson, 2002; Harvey et al., 2022), my results can guide the selection of managers for these teams, as well as the design of the managerial role itself. Indeed, firms may assign high-construal managers to those teams that need to develop sensing capabilities, and design decision-making rights and processes, roles and responsibilities, or key performance indicators and metrics that engender the required interdependence in their managers. Firms have had limited success in assigning sensing responsibilities to a handful of individuals (Nochur and Allen, 1992), and I hope that my study can help them do better.

### Limitations and directions for future research

My research has potential limitations that offer promising directions for future research. First, although I draw from the relational view of role-modeling (Harvey, 2012; Myers, 2018) in examining the moderating role of manager tenure, I stopped short of measuring and testing the frequency and quality of the communication process that occurs between managers and their teams. Hence, future research could be designed in a way that enables more direct testing of the mechanism behind this proposition. Other mechanisms may also be at play in the scaling of manager cognition to team behavior, and I cannot rule them out with my study. For instance, people’s construals can be communicated via linguistic abstraction, with consequences for advice-taking (Reyt et al., 2016) and interpersonal perception (Wakslak et al., 2014). It is possible that managers communicate their thinking style to their team via linguistic abstraction, or it could be a simple modeling of behavior. More research is needed to separate the two and further understand this mechanism. Furthermore, I cannot discard all potential sources of influence on environmental scanning behavior outside of the manager-team link. Although I control for key team and manager factors, considering staffing activities and more features of the team task could provide additional confidence in the results of my study.

Another limitation concerns the correlational nature of the data. My model confers a causal quality on the relationships I examined that cannot be inferred from correlational data (Stone-Romero and Rosopa, 2004). Although the data were collected at different times and from different sources, only an experimental research design could allow me to test the causal nature of the relationships among the different variables that I studied. To help alleviate this concern, I tested all other possible models from my set of variables,^2^ none of which provided a better fit to my data than my theorized model. Even if any had done so, I am unaware of any theories that would support a different structure, and this should remain the cardinal guiding principle to specify a particular causal sequence (Mathieu and Taylor, 2006). Nevertheless, future research should aim to replicate our findings in a more controlled, experimental setting.

Finally, the generalizability of my findings may be limited because of the type of firm in which the teams that I studied are embedded: a relatively large firm in the mortgage industry. While results have been mixed (Fainshmidt et al., 2016), technological and market dynamism may influence the development of dynamic capabilities (Zahra et al., 2006). Moreover, while teams found in large-scale firms can aim to become more innovative, other types of teams (startup teams composed of entrepreneurs, for instance) are likely required to take on more difficult challenges and develop newer competencies while negotiating greater uncertainty and managing greater risk (Lazar et al., 2020). Such teams may engage differently in environmental scanning. Specifically, entrepreneurial teams usually form endogenously—that is, their members self-select, instead of being exogenously assigned (Forbes et al., 2006). In this context, managers’ cognition may have a different impact on team behavior. This points to exciting avenues for future research, especially on developing a more comprehensive view of the antecedents of environmental scanning in teams. Nevertheless, my sample included the types of teams that can be found in most large firms (e.g. business units and organizational functions). Therefore, I emphasize that my findings offer highly relevant implications for teams with habitual routines that can limit their ability to engage in environmental scanning (Gersick and Hackman, 1990).

## Conclusion

Searching for market and technological information in the environment is at the foundation of sensing opportunities and threats (Helfat and Peteraf, 2015; Teece, 2007). My study places great emphasis on managerial cognition as the source of this key behavior and shows how it can scale to the team level in firms. My theoretical rationale and empirical findings help extend and enrich theory on dynamic managerial capabilities (Helfat and Martin, 2015) and, more broadly, the microfoundations of dynamic capabilities (Harvey et al., 2022). I hope that this will stimulate further interest in understanding which managers and teams tend to support sensing capabilities, and when and how they can do so most effectively for firms to achieve superior performance.

## Appendix 1

**Table 4.** Latent variables of the study.

Construct/Source	Prompt/Items/Scale
*Manager Construal* (α = .87) Adapted from Reyt and Wiesenfeld (2015)	*Prompt*: Imagine yourself performing the following work activities and indicate on the continuum (the verbal descriptions represent endpoints) the description that best describes each activity for you. *Items* • Preparing a report * “Getting things done”—“Telling someone what to do” • Obtaining information from someone “Asking relevant questions”—“Gaining knowledge” • Assigning work to someone “Telling someone what to do”—“Getting things done” • Communicating information to someone “Sending an email or talking to someone”—“Keeping someone informed” • Attending a meeting “Being present and paying attention”—“Staying up to date” • Developing a procedure * “Increasing work efficiency”—“Writing down step-by-step instructions” • Hiring someone “Interviewing candidates”—“Maintaining staff level” • Developing a budget “Listing expenses and revenues”—“Managing funds” • Evaluating someone’s performance “Reviewing the quality of work”—“Providing feedback” *Scale*: Low- (1) and high-level (7) activity descriptions are opposite anchors of seven-point scales
*Manager Interdependence* (α = .80) Nadler et al. (1975)	*Prompt*: Considering your current management position, indicate how much you agree or disagree with the following statements. *Items* Managers from different units do not need each other to make decisions * It is necessary for managers from different units to work together to get the job done It is in my best interest for other managers to perform well What other managers do affects what I can do *Scale*: 1 (strongly disagree) to 7 (strongly agree)
*Manager Environmental Scanning* (α = .86) Adapted from Ancona and Caldwell (1992a, 1992b)	*Prompt*: Considering your current management position, indicate how much you agree or disagree with the following statements. *Items* I look for what competing firms are doing I scan the environment for market ideas/expertise I collect technical information/ideas from individuals outside my firm I scan the environment for technical ideas/expertise *Scale*: 1 (strongly disagree) to 7 (strongly agree)
*Team Environmental Scanning* (α_team_ = .89) Ancona and Caldwell (1992a, 1992b)	*Prompt*: Considering yourself and your fellow team members, indicate how much you agree or disagree with the following statements. *Items* We look for what competing firms are doing We scan the environment for market ideas/expertise We collect technical information/ideas from individuals outside my firm We scan the environment for technical ideas/expertise *Scale*: 1 (strongly disagree) to 7 (strongly agree)

* Items that were reverse-scored.

## Notes

1. It is very common for studies of work teams to not meet that threshold. For instance, between 2018 and 2022, *Journal of Organizational Behavior*—a key outlet for team research—has published 25 team studies that use team-level measures with ICC(2) values in the .50 range or lower.

2. Notably, I tested a structural equation model without *manager environmental scanning* and *manager tenure*. Model fit is not improved (Δχ^2^ = 118.48, Δdf = 105, p = .174). Results from this model show no direct relationship between *team environmental scanning* and *manager construal level* (β = .141, p = .186). Although my empirical evidence calls for replications to establish causal order, results from this alternative model provide further support for my hypothesized model.
